# Fungal Biofilms and Polymicrobial Diseases

**DOI:** 10.3390/jof3020022

**Published:** 2017-05-10

**Authors:** Caroline B. Costa-Orlandi, Janaina C. O. Sardi, Nayla S. Pitangui, Haroldo C. de Oliveira, Liliana Scorzoni, Mariana C. Galeane, Kaila P. Medina-Alarcón, Wanessa C. M. A. Melo, Mônica Y. Marcelino, Jaqueline D. Braz, Ana Marisa Fusco-Almeida, Maria José S. Mendes-Giannini

**Affiliations:** 1 Department of Clinical Analysis, School of Pharmaceutical Sciences, São Paulo State University (UNESP), Araraquara SP 14800-903, Brazil; carolbarceloscosta@gmail.com (C.B.C.-O.); napitangui@hotmail.com (N.S.P.); haroldocdoliveira@gmail.com (H.C.d.O.); liliscorzoni@yahoo.com.br (L.S.); magaleane@hotmail.com (M.C.G.); kaylabiotech@gmail.com (K.P.M.-A.); wanessamelobio@yahoo.com.br (W.C.M.A.M.); monicayonashiro@yahoo.com.br (M.Y.M.); jaqderissi@hotmail.com (J.D.B.); almeidaf@fcfar.unesp.br (A.M.F.-A.); 2 Department of Physiological Sciences, Piracicaba Dental School, University of Campinas (UNICAMP), Piracicaba SP 13414-018, Brazil; janasardi@gmail.com

**Keywords:** fungal biofilms, polymicrobial biofilms, resistance, omics approaches, drug discovery, drug combination, in vitro techniques, in vivo techniques

## Abstract

Biofilm formation is an important virulence factor for pathogenic fungi. Both yeasts and filamentous fungi can adhere to biotic and abiotic surfaces, developing into highly organized communities that are resistant to antimicrobials and environmental conditions. In recent years, new genera of fungi have been correlated with biofilm formation. However, *Candida* biofilms remain the most widely studied from the morphological and molecular perspectives. Biofilms formed by yeast and filamentous fungi present differences, and studies of polymicrobial communities have become increasingly important. A key feature of resistance is the extracellular matrix, which covers and protects biofilm cells from the surrounding environment. Furthermore, to achieve cell–cell communication, microorganisms secrete quorum-sensing molecules that control their biological activities and behaviors and play a role in fungal resistance and pathogenicity. Several in vitro techniques have been developed to study fungal biofilms, from colorimetric methods to omics approaches that aim to identify new therapeutic strategies by developing new compounds to combat these microbial communities as well as new diagnostic tools to identify these complex formations in vivo. In this review, recent advances related to pathogenic fungal biofilms are addressed.

## 1. Introduction

Biofilm formation by microorganisms, particularly bacteria, has been widely studied in recent years. This form of growth prevails in nature compared to planktonic or free cells, and is a cause of concern mainly in the clinic because of increased resistance to antimicrobials and environmental conditions [1,2,3,4]. Biofilms formed by pathogenic fungi have gained attention in recent years and several species among filamentous, yeast, and dimorphic fungi have been described as capable of developing into communities [4,5,6,7,8]. This review aims to discuss the development of biofilms formed by yeast and filamentous fungi, interactions among polymicrobial communities, resistance to commercially available antifungals, and aspects of in vitro and in vivo methodologies and models.

## 2. Yeasts and Filamentous Fungi Biofilms

Biofilms are sessile microbial communities that strongly adhere to surfaces and to each other and are protected by a polymeric extracellular matrix (ECM) composed primarily of polysaccharides [9,10,11,12]. Cells here exhibit increased resistance and different phenotypes compared to planktonic or free cells and are associated with the persistence of infections [4,13].

Pathogenic fungi can also adhere to abiotic surfaces such as prostheses and catheters; in particular, yeasts take advantage of this condition to gain access to blood circulation, reaching the internal organs of patients. This is alarming, as disseminated fungal infections have a high mortality rate [14]. 

**Figure 1:**
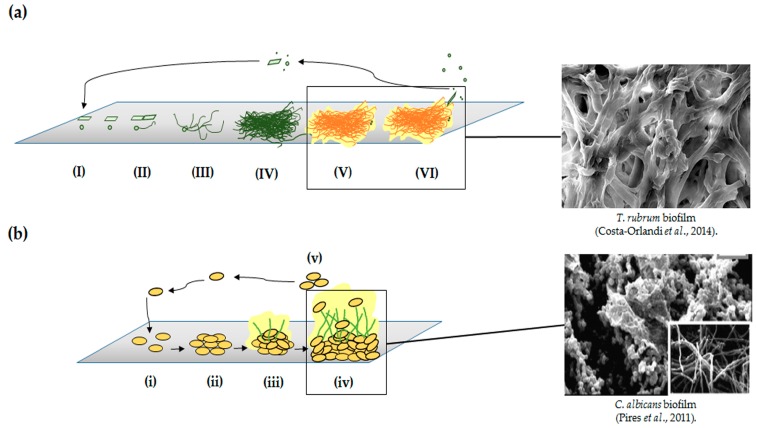
Models of biofilm development in filamentous fungi (**a**) and *C. albicans* (**b**). The stages of development are similar, although the morphology and number of stages are different. In the first model (**a**), six stages were proposed by Harding et al. [12]: (**I**) adsorption, (**II**) active attachment, (**III**) first formation of microcolony through germination and/or monolayer development, (**IV**) mycelial development, (**V**) biofilm maturation, and (**VI**) dispersion of conidia and/or arthroconidia. The second model corresponds to classical *C. albicans* biofilm development (**b**) which includes five stages, such as in bacteria: (**i**) adsorption, (**ii**) adhesion, (**iii**) microcolony formation, (**iv**) mature biofilm, and (**v**) dispersion. Modified from Harding et al. [12]. *T. rubrum* mature biofilm Costa-Orlandi et al. [7]; Pires et al. [18].

Both yeast and filamentous fungi can form biofilms; however, studies of filamentous fungal biofilms are limited compared to those of yeasts [12,15]. According to Harding et al. [12], this is because for some time the biofilms formed by filamentous fungi did not fit the previous definitions of biofilms related to bacteria. Thus, the authors proposed a model for biofilm formation by filamentous fungi, suggesting that, despite the distinct morphology, this model was similar to bacterial and yeast biofilm development. The stages of development of filamentous fungi biofilms are described in Figure 1a and include propagule adsorption (I), involving contact of spores, hyphal fragments, or sporangia to a surface; active adhesion (II), in which adhesins are secreted by spores during germination and other reproductive structures; first microcolony formation (III), which involves elongation and hyphal branching, forming a monolayer with the production of extracellular matrix; second microcolony formation or initial maturation (IV), in which compact hyphae networks form in three dimensions, covering by an extracellular matrix, and formation of water channels; final maturation (V), in which fruiting bodies and other survivor structures are formed depending of the fungi; and, finally, the dispersion or planktonic phase (VI), in which conidia and/or hyphae fragments are released, beginning a new cycle. Another peculiarity of filamentous fungi is the secretion of small proteins known as hydrophobins. These proteins are involved in the adhesion of hyphae to hydrophobic surfaces and may be involved in biofilm formation [12,16].

Regarding yeasts, *Candida albicans* is the most studied model of biofilm formation and shows distinct phases of development that are similar to those of bacterial biofilms [3,12,17]. The development process involves fewer stages of development compared to filamentous fungi and includes the adsorption of yeast cells to a surface (i); followed by initial adhesion (ii), formation of basal layers of yeast with early development of hyphae and extracellular matrix (iii); biofilm maturation containing a significant number of yeast, hyphae, pseudohyphae, extracellular matrix, and water channels that allow the movement of nutrients (iv), and cell dispersion (v) (Figure 1b) [12].

In recent years, studies correlated to fungal biofilms have increased considerably and several species have shown the ability to form these communities. *Paracoccidioides brasiliensis* is a dimorphic fungus responsible for paracoccidioidomycosis, a systemic mycosis endemic in Latin America. Sardi et al. [8] characterized the biofilms formed by this fungi in the yeast phase and found that in vitro community formation was associated with increased gene expression of adhesins and enzymes such as GP43, enolase, GAPDH, and aspartyl proteinase and decreased in phospholipase expression.

*Histoplasma capsulatum* biofilm was first described by Pitangui et al. [6]. This fungus also features thermal dimorphism and is the cause of histoplasmosis, a respiratory and systemic mycosis whose evolution depends on the survival and replication of yeast in alveolar macrophages. Therefore, the authors investigated the biofilm formation of two clinical isolates in vitro, as well as their adhesion and internalization to pneumocytes.

Dermatophytes are fungi that invade keratinized tissues producing dermatophytosis, one of the most common dermatomycoses in human and animals [19,20]. Among dermatophytosis, onychomycosis often relapses and involves long, sometimes ineffective treatment. Given this context and the hypothesis of Burkhart et al. [21], which states that biofilm formation by dermatophytes can explain dermatophytomas, Costa-Orlandi et al. [7] confirmed in vitro biofilm formation by two of the most prevalent species worldwide: *Trichophyton rubrum* and *T. mentagrophytes*.

With respect to *Histoplasma*, *Paracoccidioides*, and *Trichophyton*, other studies are being carried to characterize these biofilms, either to correlate the communities to the greater resistance to antimicrobials or select probable biomarkers using “omics” approaches (unpublished data).

Additionally, with respect to pathogenic fungi, biofilms formed by *Candida* spp. have been studied since the mid-1990s. In vitro experiments [3,18,22,23,24,25,26,27,28] are predominant compared to in vivo experiments [29,30] and confirmed the heterogeneity of these biofilms composed of dense layers of yeast blastopores, hyphal, pseudohyphae, and ECM [31,32]. Several genes are involved in the adhesion, ECM production, quorum sensing, and morphogenesis of biofilms, particularly in *C. albicans* [15,33,34]. In addition, genetic analysis confirmed that both yeasts and hyphae have unique roles in biofilm formation by this species [34]. Paramonova et al. [35] showed that most filamentation is directly related to increasing the compressive force of biofilms, which makes them more resistant to adverse conditions such as vortexing and sonication. In an study of non-*Candida albicans Candida*, Silva et al. [36] analyzed the differences regarding the formation, morphology, and composition of the ECM of biofilms formed by *C. glabrata*, *C. parapsilosis*, and *C. tropicalis*. Regarding the morphology, some biofilms of *C. parapsilosis* were composed of both yeast and pseudohyphae, although biofilms formed by other isolates were composed of only yeast cells. Finally, in *C. tropicalis*, most biofilms were composed only of yeast cells, with few exceptions showing long hyphal filaments, while *C. glabrata* biofilms contained only yeast cells. With respect to the matrix composition, biofilms showed different amounts of carbohydrates and proteins in the three species tested.

*Aspergillus* spp. are saprophytic and opportunistic fungi involved in several biotechnological processes because they secrete enzymes, proteins, and metabolites and are involved in severe superficial and systemic pathologies [37,38,39]. Aspergillosis is considered the second major cause of nosocomial infection after *C. albicans* and shows a high mortality rate [39,40]. In immunocompromised or immunocompetent individuals with previous pulmonary cavities, these fungi may cause aspergilloma, invasive pulmonary aspergillosis, allergic bronchopulmonary aspergillosis, and even systemic dissemination [38,39,41]. Aspergilloma is a fungal mass showing characteristics of biofilms [40]. As with *Candida* biofilms, the biofilms formed by these filamentous fungi have been extensively studied in recent years and can develop on abiotic surfaces [5,40,42]. A study by Mowat et al. [5] showed that these biofilms reached maturation in 24 h. At maturation, the biomass density was increased and channels developed between hyphae to allow the passage of fluids and nutrients [40,43]. The ECM is composed of α-1,3-glucans, melanin, hydrophobins, galactomannan, monosaccharides, polyols, and antigens [40,44]. 

Additionally, biofilms formed by several other fungi have been studied including those formed by *Cryptococcus* spp. [45,46,47,48]; *Malassezia* spp. [49]; *Trichosporon* spp. [50]; *Fusarium* spp. [51,52,53]; *Scedosporium* spp., *Lomentospora prolificans* [54]; and *Coccidioides* spp. [55], among others.

## 3. Polymicrobial Biofilms

Microbes rarely exist in single-species planktonic forms [56]. Most microorganisms live in complex communities, known as polymicrobial biofilms [11,57]. Similarly to most communities, biofilms are multicultural and well-engineered [58]. Interactions within these biofilms can be mutualistic, commensalistic, or antagonistic and microorganisms have evolved highly defined responses to sense and adapt to neighboring species [59,60].

In terms of human health, polymicrobial biofilms are prevalent throughout the human body, both during healthy and disease conditions [58]. However, the clinical concern regarding the synergies of polymicrobial biofilms is that the infection will be more severe and recalcitrant to treatment [61]. Microbial synergy is a cooperative interaction between two or more species that produces an effect not achieved by an individual species alone [56,62,63,64,65]. These synergistic interactions are more severe than infections with individual microorganisms [62], leading to increased antimicrobial resistance and prolonging the time necessary for host recovery [58].

Genetic diversity of biofilm communities increases the fitness of the residing community, making the species better able to survive to environmental pressures [61,66], resulting in accelerated growth [67], increased stress resistance [68,69], immune evasion [70,71,72,73], passive resistance [74], and metabolic cooperation [75,76]. In bacteria, it was demonstrated that this occurs because of an expanded gene pool that can be shared within the residents of the biofilm community [66,77,78].

Infections related to polymicrobial biofilms are most frequently observed in the urinary tract, lung, inner ear, urinary tract, oral cavity, wounds, and abiotic devices [66,79]. Biofilms at these sites can potentiate infection and induce a chronic inflammatory state, resulting in collateral damage to host tissue. This fact and the structure of biofilms help to protect microbes from antimicrobials, host immunity, and environmental factors [66].

In recent years, through the development of more sophisticated technologies, the understanding of the importance of polymicrobial infection in human fungal disease has increased. Fungal–polymicrobial interactions are important in a variety of disease states and niches including respiratory system infections, formation of dental plaque, invasive disease, skin and mucosal infections, and bloodstream infections [80].

Recent studies have shown that *Candida* rarely exists as monospecies and can colonize mucosal surfaces and prosthetic materials, such as dentures and catheters, throughout the human body. In addition, polymicrobial communities consisting of aggregates of other fungi and bacteria are highly prevalent and clinically important [81,82]. 

Oral candidiasis is one of the most well-defined fungal biofilm infections and is characterized by complex biofilms, which interact with bacteria and the host [81,83,84]. The relationship between *Candida* and streptococci is generally considered to be synergistic, where a streptococcal infection interacts with the hyphal filaments of *Candida* via cell surface adhesin SspB interacting with the hyphal cell wall protein Als3 [82,85,86,87]. Some studies showed that bacteria can enhance biofilm formation and the pathogenicity of *C. albicans* [82,88]. In this interaction, streptococci provide *Candida* with nutrients from the salivary pellicle [89], while *Candida* promote the survival of streptococci by lowering oxygen tension levels to those more acceptable for streptococcal growth and providing nutrients to stimulate bacterial growth [81].

Biofilms composed of *Staphylococcus aureus* and *Candida albicans* have been widely studied, as these two organisms are often found together in different types of infections, where they show enhanced virulence and resistance upon co-infection of hosts [64]. *Staphylococcus aureus* and *Candida* spp. are two of the most prevalent bloodstream pathogens and are responsible for severe morbidity and mortality in hospitalized patients. There is some evidence that they are commonly associated as co-infecting organisms [90,91,92,93]. In addition to the bloodstream, *C. albicans* and *S. aureus* have been co-isolated from various mucosal surfaces including the vaginal and oral mucosa in a biofilm mode of growth [91,94,95,96]. According to Peters et al. [91], a proteomics approach to identify proteins upregulated during a *C. albicans–S. aureus* interaction demonstrated that both species could induce a stress response upon their initial interaction, particularly when *Candida* was still in the yeast form. However, during biofilm maturation, some genes may be downregulated as a survival strategy, enabling survival within the host.

Moreover, although poorly studied, there are some reports of *Candida–Candida* mixed biofilms. Coco et al. [97] reported the isolation of *C. albicans* and *C. glabrata* co-infection from patients with severe inflammation and hypothesized that pathogenic synergy occurred. Further studies confirmed this synergy, in which *C. albicans* appeared to assist *C. glabrata* in invading in vitro reconstituted epithelium [98]. In another model corresponding to the human vaginal epithelium, *C. glabrata* in combination with *C. albicans* caused significant tissue damage compared to *C. glabrata* alone [99].

Recently, Martins et al. [26] reported the in vitro formation of a mixed biofilm containing *C. albicans* and *C. rugosa*, an emerging fungal pathogen found in Latin America, particularly in Brazil. *Candida rugosa* shows a lower susceptibility to fluconazole, amphotericin B, and echinocandins and is frequently found in elderly patients with a capacity to form biofilm [100,101,102,103,104,105]. Kirkpatrick et al. [106] also described mixed biofilms formed by *C. albicans* and *C. dubliniensis* and showed that *C. albicans* had a distinct competitive advantage over *C. dubliniensis* under planktonic growth conditions, while under biofilm growing conditions, *C. dubliniensis* was able to better withstand the rigorous competitive pressures from *C. albicans*.

Other fungi species were also described in vitro as part of polymicrobial biofilms. *Aspergillus fumigatus* and other species of *Aspergillus* are commonly found to co-colonize with *Pseudomonas aeruginosa* in the lungs of cystic fibrosis (CF) patients, open skin wounds, and cardiac implants [107]. Manavathu et al. [108] demonstrated in vitro that polymicrobial CF patient airway infection with *P. aeruginosa* and *A. fumigatus* produced mixed microbial biofilm with structural and functional characteristics differing from those of monomicrobial biofilms, which is a serious clinical problem in CF patients and other patient groups prone to airway infection with *P. aeruginosa* and *A. fumigatus*. Zheng et al. [107] demonstrated that phenazine-derived metabolites produced by *P. aeruginosa* can act as signals that affect *A. fumigatus* and *A. nidulans* development, shifting from weak vegetative growth to induced asexual sporulation (conidiation) along a decreasing phenazine gradient, affecting biofilm formation.

Numerous studies have described co-infections of fungi and bacteria in different diseases. As an example, the cystic fibrosis lung is a major site of polymicrobial infections, with bacteria such as *P. aeruginosa*, *S. aureus*, *Burkholderia cepacia*, *Acinetobacter baumanii*, *Haemophilus influenzae* mixed with *C. albicans*, *A. fumigatus*, and *Scedosporium* species [80]. However, additional studies are needed to understand how these interactions occur and to determine the involvement of biofilm communities in these infections.

At other sites of infections, different polymicrobial interactions between fungi and bacteria have been described. As an example, *Candida* interacting with *Streptococcus* and *Lactobacilli*; *Porphyromonas gingivalis* at oral sites; *Candida*, *Aspergillus*, *Mucorales*, and *Fusarium* with *Pseudomonas* and *Staphylococcus* in burn wounds and trauma sites; *Candida* and *Cryptococcus* with a wide range of Gram-negative and Gram-positive bacteria in the lower reproductive tract; *Candida* with Gram-positive and Gram-negative bacteria (typically *Staphylococcus* spp.) and interactions between dermatophyte species in the cutaneous site and vascular catheters, Enterobacteriaceae and *Enterococcus* spp. with *Candida* spp. in intra-abdominal site and *Pseudomonas* spp. and, finally, Enterobacteriaceae, *Escherichia coli* and *Enterobacter faecalis* with *Candida* spp. in the urinary tract [80,109]. Gastrointestinal tract and gut interactions between *Candida* spp. and *E. coli*, *Helicobacter*, *Serratia marcescens*, and *Salmonella enterica* subsp. *enteric*a serovar *Typhimurium* have also been reported [80,110].

The increasing number of fungal infections associated with the increase in descriptions of fungal co-infections with bacteria and other microorganisms reveal the importance of further studies of the mechanisms and consequences of polymicrobial biofilm formation.

## 4. In Vitro Methods to Study Biofilms

### 4.1. Conventional Methods

Biofilm-associated infections are a serious public health problem because this microenvironment can reduce the efficacy and susceptibility to antifungal agents and avoid the host immune response. Thus, the development of biofilms has been extensively studied. Several in vitro methods are used to evaluation biofilm progression. The main characteristics assessed are cell adhesion, production of ECM, biofilm architecture, mechanism of drug resistance to antifungal agents, cell phenotypes, and for certain fungal species such as *Candida albicans*, yeast-to-hyphae transition [17,111,112]. The methods described below are employed in studies of both biofilms formed by filamentous fungi and those formed by yeasts.

Colorimetric assays are commonly used in studies of the development and susceptibility of biofilms to antifungal drugs. Hawser and Douglas [24] were pioneers in studies of fungal biofilm formation employing the methyltetrazolium assay (MTT (3-[4,5-dimethylthiazol-2-yl]-2,5-iphenyltetrazolium bromide)). MTT is a yellow soluble salt; in the presence of metabolic activity, the salt is reduced to an insoluble purple formazan crystal. The same authors concluded that the converted MTT was highly correlated with biofilm dry weights and could be applied to evaluate fungal biomass. Since then, this technique has been widely accepted [111,113,114,115]. A disadvantage of this technique was reported by Manavathu et al. [108] when using MTT to determine the effects of antimicrobials on polymicrobial biofilms of *A. fumigatus* and *P. aeruginosa*. According to the authors, although this method was useful for monitoring monospecies biofilms of *A. fumigatus*, it was difficult to differentiate the contribution of each microorganism in the reduction of MTT compound when mixed biofilm was evaluated. 

XTT (2,3-*bis*(2-methoxy-4-nitro-5-sulfophenyl)-5-[(phenylamino)carbonyl]-2*H*-tetrazolium hydroxide) is another tetrazolium salt employed to analyze biofilm development and drug susceptibility. This salt is converted to water-soluble colored formazan salt in the presence of metabolic activity by cellular effectors, such as mitochondrial dehydrogenases [111,116]. Among colorimetric methods, XTT has been the most widely used in recent years [5,6,7,8,46,48,117,118]. XTT is the method of choice employed in susceptibility tests [114]. Compared to MTT, the XTT technique is advantageous because the amount of formazan obtained as a product can be measured directly in the supernatant, while the MTT requires another step involving cell lysis, in which cells must be treated with dimethyl sulfoxide before optical density measurement [111]. However, there are some disadvantages to the use of XTT. Studies of the growth and metabolism of planktonic cells and biofilms of *Candida* reported that although there the colorimetric signal is proportional to the number of cells, there may be variations when comparing different strains of *Candida*. The authors stated that there may not be a linear relationship between the number of microorganisms and colorimetric signal, suggesting that this quantification is only valid after constructing a standard curve for each concentration of tetrazolium salt used. In addition, they also stated that significant salt retention may occur when comparing microorganisms in planktonic or biofilm forms. 

In addition to MTT and XTT, other assays in microtiter plates for susceptibility testing and biofilm characterization have been explored, such as Alamar blue/resazurin [119,120,121]; safranin [7,42,122]; crystal violet [7,31,119]; Alician blue [123,124], and DMMB (1,9-dimethyl methylene blue) [119,125] (Table 1). Furthermore, scanning and transmission electron microscopes and confocal microscopy have been used to study and detect biofilm cells and ECM [6,7,8,126,127,128].

Scanning electron microscopy (SEM) is a technique in which a sample is prepared by fixation, dehydration, and drying, and the image is processed after coating the samples with a conductor such as gold or carbon under a high vacuum. However, drying and dehydration can alter biofilm morphology because of ECM collapse. Furthermore, artifacts can alter the images. Alternatively, environmental SEM has emerged as a method of choice because the biofilm can be observed without fixation and dehydration and the vacuum is moderate, preserving the morphology and structures of the surfaces [128,129].

Transmission electron microscopy (TEM) is also employed to visualize biofilm architecture. The preparation for TEM is similar to that of SEM. However, in this method, the biofilm is embedded in a resin that allows the ECM to remain stable, unlike in SEM. One disadvantage of TEM is that it is not possible to visualize the biofilm topography [128].

Confocal laser scanning microscopy (CLSM) is another tool used to analyze the biofilm three-dimensional (3D) architecture and thickness. In addition, it is possible to verify the presence of macromolecular compounds, such as polysaccharides, proteins, nucleic acids, and lipids [111,127]. Another advantage is that CLSM can be employed with or without fluorescence or with fluorescence in situ hybridization (FISH) to evaluate alterations of specific compounds of fungal populations over time and spatial relationships [130].

Among other microscopy techniques, which are often costly, scanning transmission X-ray microscopy is associated with near-edge X-ray absorption spectroscopy [127]; CLSM in combination with Raman microscopy (RM) [131]; episcopic differential interference contrast microscopy with and without fluorescence; Hoffman modulation contrast microscopy; and atomic force microscopy [132] have been used to examine biofilms in situ.

### 4.2. High-Throughput “Omics” Technologies in Biofilms Research

Microbial biofilms involve complex regulatory systems that can be elucidated using “omics” technologies. “Omics” approaches are powerful tools for quantifying differentially global variations between two different biological conditions on a transcriptomic, proteomic, and/or metabolomics scale and targeting for the discovery of novel therapeutics and/or biomarkers in the cell host–fungi interaction [135].

In general, these high-throughput “omics” tools compare two biological systems based on the abundance of messenger RNA transcripts (transcriptomic), proteins (proteomic), and other biomolecular components (metabolomics). These tools began in the so-called post-genomic era and were developed to overcome the limitations associated with previous genomic investigations of organisms in question. Genomic sequencing reflects genetic information through techniques that identify the correct nucleotide sequence in the organism's genome. In this context, aspects such as sequence, number, and syntenia of the genes contained in the nucleus of a cell remain static during the cell cycle; however there is a dynamic equilibrium between: (a) gene transcription, (b) protein translation, and (c) production of metabolic byproducts according to the biological situation to which a cell is subjected, thus defining different transcriptomes, proteomes, and metabolomes for the same cell throughout its cellular differentiation [136].

Therefore, transcriptomics, proteomics, and metabolomics analyses can be employed to determine differences between the transcriptional, translational, and metabolic signatures of a microorganism in biofilms and in planktonic growth. This field of research revealed new concepts; in this context, Azevedo et al. [137] suggested a new trend, so-called “biofomics,” an omics approach to the field of biofilms. The goal of this approach is to gather in an on-line database a large set of omics data generated from studies of a microorganism's ability to adhere to surfaces, communicate with its neighbors, and form biofilms. Such collected data would be freely available to the scientific community and identify a unique biofilm “signature” including important information such as environmental, physiological, and mutational factors that affect the ability of a microorganism to develop biofilms. This may positively impact systems biology and consequently the development of a new diagnostic tool and/or therapeutic for resistance development.

Recent studies using high-throughput omics methods to evaluate biofilms have revealed important information for the development of new therapies for biofilm-based fungi infections. For instance, a recent study showed that *C. albicans* constitutes the most prevalent and pathogenic species among all *Candida* species related to *Candida* bloodstream infections because of the species’ ability to form robust biofilms. Rajendran et al. [138] showed that clinical isolates of candidemia may be stratified as high- or low-biofilm formers (high biofilm formers (HBF) and low biofilm formers (LBF), respectively), resulting in a heterogeneous biofilm phenotype; according to its classification, this directly determines clinical outcomes and mortality. These authors found significant differential gene expression between *C. albicans* LBF and HBF by comparative RNA-Seq analysis on pre-characterized clinical isolates and emphasized the importance of the aspartate aminotransferase pathway in biofilm formation, which may be exploited as a potential target. 

Concomitantly, according to Otto et al. [139], modern proteomic techniques provide a detailed description of the protein inventory of a cell and consequent changes in protein levels through relative or absolute quantification. In this sense, pathogenic fungi such as *Candida* species and *C. neoformans* have differential proteomes between planktonic and biofilm cultures as established by several authors [28,140,141,142,143]. However, features of biofilms formed by other pathogenic fungi, such as *H. capsulatum*, *P. brasiliensis*, and dermatophytes remain unknown. The findings of our group revealed a substantial difference in the protein profile between *H. capsulatum* yeasts structured in biofilms and planktonic growth, with more than 40 differentially expressed proteins identified by mass spectrometry. Such proteins are involved in the metabolism of amino acids, nuclear proteins, and translated proteins [10] and should be explored for the development of safer and more effective drugs. In addition, Pires et al. [28] used a proteomics approach to compare the protein expression of *C. orthopsilosis* planktonic and biofilm forms. According to these findings, differentially expressed proteins were linked to several suitable mechanisms that adjust the catalytic properties of the enzymes. 

Additionally, an analysis of the metabolic signature of *C. albicans* during biofilm development has been published by Zhu et al. [27] who conducted gas chromatography-mass spectrometry and identified 31 differentially produced metabolites between the biofilm and planktonic cells. This study showed even that trehalose is involved in the formation of *C. albicans* biofilms, as the lack of this metabolite resulted in abnormal biofilm formation and increased the susceptibility of biofilms to the antifungal amphotericin B and miconazole. 

In fact, advances in high-throughput DNA and RNA sequencing and mass spectrometric quantification of proteins and metabolites, combined with computational tools, have enabled researchers to obtain large amounts of data generated in these “omics” projects. Various research groups have integrated “omics” datasets. This combination represents much more than the sum of each “omics” analysis, generating data related to interactions that can occur among all classes of molecules in a cell. Thus, an understanding of interactions on different levels, such as genomic, epigenomic, transcriptional, proteomic, post-translational modification, and metabolic, enables the mapping and elucidation of peculiar information regarding the behavior of a cell during the same biological process [144].

Muszkieta et al. [135] integrated three different “omics” methods, microarray, RNA sequencing, and proteomics analysis, to compare the different transcriptomics and proteomics signatures of *A. fumigatus* biofilms. According to the authors, although utilization of several integrated “omics” methods remains challenging, such a combination provides potential information that answers important biological questions. Therefore, comparison and integration of “omics” approaches in different levels can provide exciting information for characterizing the microbial biofilm signature, such as formation potential, physiological activity, and structure. 

In conclusion, although there are challenges in biofilms research using “omics” technologies, these approaches, whether isolated or integrated, provide promising results for future studies targeting novel potential therapeutics and/or biomarkers for the diagnosis of fungi biofilm-associated infections.

## 5. In Vivo Models to Study Fungal Biofilms

Biofilm formation provides protection to fungal cells (stress, immune system, antifungal drugs). Recently, different techniques based on morphologic and biochemical characterization have become available for evaluating biofilms in vitro. However, biofilms must be studied using complex organisms. The use of in vivo models provides important information regarding the influence of host factors on biofilm formation. Some host factors have been found to affect biofilm formation. (1) Flow conditions: some fungi species can form biofilm under static conditions, while other species produce biofilm under different flow conditions, requiring the production of a large amount of ECM, reflecting increased antifungal resistance [145,146,147]. (2) Substrate: surfaces with certain topography and hydrophobicity conditions favor biofilm formation to provide good adhesion conditions. Medical devices are carefully designed to resist microbe adhesion; however, molecules present in host fluids can promote biofilm formation in these devices. Moreover, biotic surfaces can present receptors that facilitate the cell-microorganism link [147,148,149]. (3) Nutrient conditions: the availability of sugar, proteins, and metal ions impact biofilm characteristics [147,150,151]. (4) Immune system: leukocytes and mononuclear cells interact with biofilm [149,152]; different antibodies also interact with biofilm [153].

The use of medical devices (catheters, dentures, and subcutaneous implants) permits the adhesion of fungal cells and formation of biofilms, making the eradication of infection difficult [154]. *Candida* spp. are among the most studied yeasts in the context of biofilms. This yeast can grow as biofilm on biotic surfaces during oral, oropharyngeal, and vulvovaginal infection [149,155]. *Aspergillus fumigatus* can form biofilm during different clinical presentations like sinusitis, pulmonary aspergillosis, and aspergilloma [38]. Based on this, models using medical devices to study in vivo biofilm were developed to better understand biofilm life cycle and treatment. Models using only biotic surfaces have also been described.

The vascular catheter model is one of the most widely used approaches for evaluating in vivo biofilm formation. This model simulates biofilm clinical infections and host conditions such as flow, nutrition, and immune system. In contrast, the surgical process for implanting the catheter is laborious and invasive [147,156]. This in vivo model can be performed in different animal such as mice, rats, and rabbits. The advantage of using mice is their low cost compared to other organisms. However, the surgical procedures are more difficult to execute because of the diameter of the vessels. The rabbit model is easier to manipulate, but more costly [156]. Microorganisms can access vascular catheters through the skin, fluids, medicines, and other avenues, forming biofilm on the intraluminal or extraluminal side of this device [157]. This model was also described in rats and reproduced architectural structures as in vitro models [158]. Martinez et al. [159] used a vascular catheter model in rats to assess the effect of catheters coated with chitosan, a crustacean exoskeleton polymer. Promising results were obtained for the inhibition of *Candida* spp. biofilm formation and cell viability decrease. The vascular catheter model using rabbits was first described by Schinabeck et al. [160] in a study of biofilm formation and treatment. Amphotericin B liposomal was found to be effective for eliminating biofilms and treating *C. albicans* infection associated with the catheter. Using the same model, Ghannoum et al. [157] tested the efficacy of micafungin against *Candida* biofilms, revealing the ability of this antifungal to eradicate the infection and inhibit biofilm growth.

Subcutaneous in vivo biofilm models possess advantages such as the ability to implant more than one device per animal. Compared to other models, the procedure is fast, less aggressive, and requires a shorter anesthesia period [29,147]. In addition, this system can mimic joint prostheses; however, the model suffers from nutrient deprivation because of inconsistent fluid irrigation [161]. A subcutaneous rat model was developed to study *C. albicans* biofilm; this avascular location of the catheters reduced the experimental procedure and the biofilm extracellular matrix was observed just two days after infection [29]. *Candida glabrata* cannot produce hypha, which is considered an essential phenomenon for *C. albicans* biofilm formation [15]. Based on this, Schinabeck et al. [160] demonstrated in a rabbit subcutaneous catheter model that *C. glabrata* formed biofilm in vivo with half the thickness of the *C. albicans* biofilm, despite that *C. glabrata* in vivo biofilm was susceptible to echinocandins but not to fluconazole.

Mucosal tissues exhibit satisfactory conditions for biofilm growth, mainly because they provide a nutrient-rich environment for microbial communities and because of the unique host–microbial interactions that can affect both the host responses and biofilm development, an advantage of this model compared to abiotic surfaces [149]. However, the recognition of many tissue infections as biofilms remains a critical process [149]. Oral candidiasis was found to commonly affect immunosuppressed patients [162]; moreover, denture stomatitis related to polymicrobial biofilm including *Candida* spp. induces mucosal biofilms. Thus, immunosuppressed animals are used to simulate this model, inoculating the fungi using a swab on the tongue or sublingually [163]. A rat model was demonstrated to be suitable for analysis of in vivo mucosal device-associated infections, particularly given the cost of the animal; moreover, rats show great potential for mimicking denture stomatitis as well as for evaluating mixed microorganism biofilm and the immune response [164]. Mouse experimental oral candidiasis was used to evaluate the effect of photodynamic therapy [165]. In another study, induction of oral candidiasis was performed in mice using a bioluminescent strain of *C. albicans* to evaluate the action of the polyphenol lichochalcone-A. Longitudinal imaging and histological analysis of mice infected with the bioluminescent strain and after treatment revealed the antifungal efficacy of lichochalcone-A [166]. An oropharyngeal candidiasis model in mouse [163] was used to demonstrate the in vivo efficacy of miltefosine, which was previously used in vitro and efficiently inhibited biofilm formation and for oropharyngeal treatment [167].

The incidence of urinary catheter-associated infection is high during hospital confinement. To study the involvement of *Candida* spp. biofilm in this infection, a urinary in vivo biofilm model was developed. Rat and mice were used as models to study *Candida* spp. biofilm characteristics as well as the importance of the biofilm in the persistence of the candiduria, biofilm antifungal efficacy, mutant phenotypes, and new anti-adhesive materials for catheters [168,169].

*Fusarium* spp. can form biofilms on contact lenses, which is a risk factor for the development of keratitis. Sun et al. [170] developed an in vivo model for contact lenses containing *Fusarium* spp. biofilm, which were applied to the mouse cornea. Important features of *Fusarium* spp. biofilm formation in vivo and the immune response were elucidated. Taking a different approach, Pinnock et al. [171] used ex vivo corneas from both rabbits and humans to study *C. albicans* and *F. solani* biofilms as alternatives to in vitro or in vivo models to study keratitis. Another approach was developed to simulate *Fusarium* spp. keratitis by direct application to the cornea without contact lenses in a murine model using fluorescent staining. This simple method enables detection of infection in early stages [172].

Despite the use of these mammalian models for studying in vivo biofilm formation and treatment, the use of invertebrate models to determine biofilm characteristics has recently been encouraged because of ethical issues related to mammalians models. The use of *Galleria mellonella*, an insect model, revealed information regarding the virulence of *Cryptococcus* spp. planktonic and biofilm cells, demonstrating that cells originating from biofilm killed *G. mellonella* larvae faster than planktonic cells [173]. The effectiveness of acetylcholine for inhibiting *C. albicans* biofilm was studied in the *G. mellonella* model [174]. In addition, the in vivo virulence of clinical strains of *C. albicans* capable of forming biofilms was evaluated in *G. mellonella*, revealing the strong virulence of biofilm-producing strains [175].

Significant advances have been made in understanding biofilms and in their therapy by using in vivo models. However, many aspects of biofilms remain unclear and in vivo studies are fundamental for understanding biofilm–host interactions and developing anti-biofilm compounds/strategies.

## 6. Physical and Molecular Resistance in Fungal Biofilms

Among the defining characteristics of biofilms are their high resistance to antimicrobial agents and production of ECM. The matrix protects and envelops the entire biofilm, providing an ideal structure for cell cohesion and adhesion [176]. The ECM also retains water and nutrients derived from matrix materials hydrolyzed by enzymes produced by microorganisms [176].

The most medically relevant function of the extracellular matrix is its ability to provide a physical barrier between biofilm cells and immune system and often drugs used for treatment [9,154,177]. Fungus biofilms were reported to be up to 1000-fold more resistant to antifungal agents than planktonic cells, but the mechanism of this resistance remains unclear [10,123]. 

**Figure 2:**
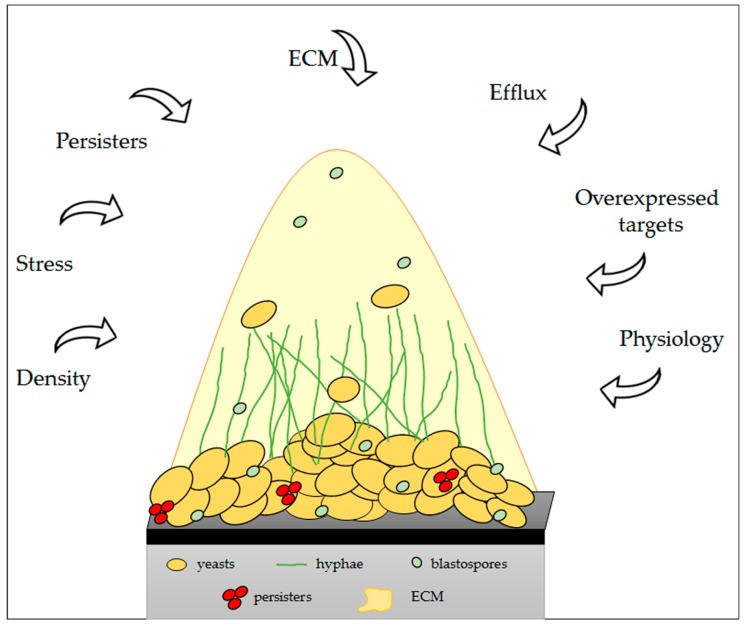
Scheme of the mechanisms and factors that promote fungal biofilm resistance, which are common to several fungi. Adapted from Ramage et al. [189].

Antifungal resistance is very complex and multifactorial. It may be inducible in response to a drug, biochemical alterations, or an irreversible genetic change resulting from prolonged exposure (Table 2). Specifically, alterations or overexpression of target molecules, active extrusion through efflux pumps, limited diffusion, tolerance and cell density (quorum sensing), and the ECM are all characterized mechanisms used by fungi to combat the effects of antifungal treatment (Figure 2). 

Planktonic cells generally depend on irreversible genetic changes to maintain a resistant phenotype, while biofilms persist because of their physical presence and population density, providing a nearly inducible resistant phenotype regardless of the defined genetic changes [10,187,188]. Further, factors including pH, temperature, oxygen availability, and other environmental stresses alter the biofilm architecture and possibly antifungal susceptibility [189].

A defining characteristic of a biofilm is its ECM, which is self-produced and may contain proteins, polysaccharides, lipids, nucleic acids, and other molecules [176,190] that can interact with each other and with the cell surface to form a robust and protective network [191]. ECM composition varies across species and even growth conditions [176]; however, the ECM composition of many biofilms remains unknown [176]. Functionally, the ECM can serve as a protective barrier against chemical and biological antimicrobial agents, including many prescribed antifungal drugs [178,190]. In some instances, the ECM can contribute to antifungal resistance by binding to antifungals, thereby preventing access to their intended target at the surface or within fungal cells [192].

Quorum sensing is related to the mechanism by which microorganisms communicate and coordinate their behavior through the secretion of signaling molecules [193,194]. Cells respond to these quorum-sensing molecules (QSMs) through the expression or repression of quorum-dependent target genes [10,195]. QSMs play a role in several mechanisms, including biofilm development, morphogenesis, and limitation of cell population, among others. They are also important during the infectious process, particularly for dissemination [194,196]. Farnesol was described for the first time in *C. albicans* by Hornby et al. [197] as a QSM. Contact between *C. albicans* and exogenous farnesol results in several responses, including activation of genes involved in drug resistance (CaFCR1 and CaPDR16) [189,198]. Another study conducted by Sharma et al. [199] demonstrated that farnesol modulated the action of drugs in *C. albicans* planktonic cells. In addition, Ramage et al. [200] concluded that farnesol inhibits hyphae development during the initial phase of biofilm formation, compromising the structure. Farnesol also affects many other microorganisms such as *S. aureus*, *S. cerevisiae*, *Aspergillus* spp., *P. brasiliensis*, and *Mycobacterium smegmatis* [201,202]. The detection of QSMs is of fundamental importance, as they have specific roles in biofilm physiology (Table 3).

Persisters represent a small subpopulation of cells that spontaneously enter a dormant, non-dividing state. When a population is treated with an antimicrobial, normal cells die, while persisters survive. When therapy is discontinued, the persistent cells can restore the biofilms, thus explaining why biofilm infections are recurrent [203,204].

The molecular mechanisms that promote antifungal resistance in fungal biofilms are not completely understood. Some studies have shown that efflux pumps contribute to azole resistance only during the early phase of biofilm formation. In addition, membrane sterol composition contributes to azole resistance, but this occurs during the intermediate and mature phases [205]. According to Soto [206], the upregulation of drug efflux pumps also causes drug resistance in several biofilm-forming microorganisms. These pumps are divided into two groups; the first is linked to ATP-binding cassette transporters encoded by CDR-genes, while the second is composed of the major facilitator superfamily encoded by MDR-genes [178]. Activation of drug efflux pumps occurs through the “expulsion” of antifungal drugs after contact with the biofilm. The increases the expression of the CDR1 and MDR1 genes, in which has been correlated to the resistance of yeast to azole drugs. Some data regarding biofilm-associated resistance have shown that the expression of efflux pump genes is increased during the first hours of biofilm formation [178].

## 7. Conclusions

Biofilm formation by saprophytic and pathogenic fungi is of great concern. Despite recent advances in the study of biofilms formed by species in the genus *Candida*, *Cryptococcus*, and *Aspergillus*, reports of biofilm formation by other species are increasing. Therefore, an understanding of the molecular mechanisms and key factors involved in establishing these infections is necessary. In addition, interactions between biofilms of polymicrobial origin and the host should be prioritized in studies, particularly those of recently described biofilms. Finally, the discovery of new treatment alternatives capable of controlling or destroying these microbial communities is essential. 

## Figures and Tables

**Table 1:** Microtiter plates assay for susceptibility testing and biofilm characterization.

Microtiter Plates Assays	Characteristics
MTT	MTT is a yellow soluble salt, which in the presence of metabolic activity, is reduced to an insoluble purple formazan crystal. This method is used to determine the metabolic activity of some microorganisms in planktonic and biofilm forms. Moreover, this method shows excellent correlation with biomass determination by dry weight. Fast and convenient [24].
XTT	Tetrazolium salt (yellow) is reduced by the activity of fungal mitochondrial dehydrogenase to formazan salt (orange), which is correlated with cell viability. It is also used to determine metabolic activity in the developmental stages of biofilms and in antifungal susceptibility tests [46,116,118]. The method is simple and, reproducible, but some disadvantages were reported by Khun et al. [116].
Alamar Blue and Resazurin	Reduction is dependent on metabolic activity. The methods are fast and simple and measurement can be conducted spectrofluorometrically or spectrophotometrically. Resazurin is the active principle of Alamar Blue. The reagents are nontoxic to humans and fungi and the method is reproducible. Good correlation with XTT assay and CFU/mL [133,134]. Used for biofilm quantification. Blue dye resazurin is converted to pink resorufin in the presence of metabolic activity. Nontoxic and soluble in water [119,120,121].
Safranin	Dye easy to use for ECM quantification Difficult interpretation; low-cost [7,42,122].
Crystal Violet (CV)	Used for biomass quantification. CV stains living and dead cells, and thus it is not indicated to verify antifungal activity in biofilms [119]. Low cost and easy [31].
Alcian Blue	Measures mass quantity of biofilm ECM [123,124].
1,9-Dimethyl Methylene Blue (DMMB)	Quantification of biofilm matrix [119,125].

**Table 2:** Resistance mechanisms associated with biofilm formation. Adapted from Mathé and Dijick [178] and Sardi et al. [179].

Resistance Mechanisms	Effect	References
Cellular density	Quorum sensing	Perumal et al. [180]; Seneviratne et al. [181].
Differential regulation drug target	Alteration in target levels; Associated with changes in target structure that make the drug unable to bind to the target.	Nailis et al. [182].
Upregulation drug efflux pumps	Antifungal is pumped out of cells and thus cannot perform its intracellular function.	Nett et al. [183]
Persister cells	Because of the dormant state of the persisters, antifungal targets are inactive.	LaFleur et al. [184]
Presence of a matrix	Specific binding of antifungals to β-1,3-glucans, a major component of the matrix, prevents antifungal agents from reaching their targets.	Al-Fattani and Douglas [185]; Mitchell et al. [177].
Diverse stress responses	Possible indirect effects through the regulation of other resistance mechanisms.	Diez-Orejas et al. [186]

**Table 3:** Role of QSMs (quorum-sensing molecules) in yeasts and dimorphic fungi. Adapted from Wongsuk et al. [194].

Organism	QSMs	Role of QSMs in Molds and Dimorphic Fungi	References
*C. albicans*	Farnesol	Inhibited hyphal development Involved in morphogenesis Inhibited biofilm formation Induced apoptosis Antifungal activity Modulated drug extrusion	Nickerson et al. [207]
			Martins et al. [208]
			Ramage et al. [200]
			Shirtliff et al. [209]
			Sardi et al. [10]
			Sharma et al. [199]
	Tyrosol	Promoted germ tube formation Stimulated hypha production during the early stages of biofilm development Antifungal activity	Alem et al. [210]
			Chen et al. [211]
			Cordeiro et al. [212]
*A. niger*	Farnesol	Inhibited conidiation Reduced intracellular cAMP levels	Lorek et al. [213]
*A. fumigatus*	Farnesol	Altered growth phenotype Perturbed cell wall	Dichtl et al. [214]
*H. capsulatum*	Farnesol	Inhibited biofilm formation Antifungal activity	Brilhante et al. [215]
*P. brasiliensis*	Farnesol	Inhibited growth Delayed the dimorphic transition Antifungal activity	Derengowski et al. [216]
